# Effectiveness of group acceptance and commitment therapy in treating depression for acute stroke patients

**DOI:** 10.1002/brb3.3260

**Published:** 2023-11-08

**Authors:** Yun‐E Liu, Jin Lv, Fang‐Zhen Sun, Jing‐Jing Liang, Ying‐Ying Zhang, Jie Chen, Wei‐Jian Jiang

**Affiliations:** ^1^ New Era Stroke Care and Research Institute The PLA Rocket Force Characteristic Medical Center Beijing China; ^2^ Department of Radiotherapy The PLA Rocket Force Characteristic Medical Center Beijing China; ^3^ The PLA Rocket Force Characteristic Medical Center Beijing China

**Keywords:** acceptance and commitment therapy, acute stroke, depression, effectiveness, nurse

## Abstract

**Objectives:**

To date, the effectiveness of acceptance and commitment therapy (ACT) for acute stroke patients has not been well recognized. The study aimed to discover the effectiveness of group‐based ACT in treating depression for acute stroke patients.

**Methods:**

We conducted a randomized controlled trial with 140 acute stroke patients with depression. The ACT intervention comprised seven sessions, of 45–60 min over 4 weeks. Data were collected pre‐ and post‐intervention and at 3‐month follow‐up, assessing depression, health‐related quality of life (HRQoL), psychological flexibility, cognitive fusion, sleep quality, and confidence.

**Results:**

Overall, 99.3% of the included patients were assessed as having mild depression. The ACT intervention significantly reduced depression in acute stroke patients in comparison with the control group post‐intervention and at 3 months (partial $\eta^{2}=.306$). Additionally ACT significantly improved HRQoL‐mental component summary, sleep quality, psychological flexibility, cognitive fusion, and confidence compared with control group.

**Conclusions:**

ACT is effective in treating acute stroke patients with depression, and the efficacy was maintained at 3‐month follow‐up.

## Graphical abstract

This study found that G‐ACT could significantly reduce depressive symptoms in patients with acute stroke, and the interventional efficacy was maintained at 3‐month follow‐up (partial Ƞ2 for this effect was 0.306), with similar benefits being observed for HRQoL‐MCS, sleep quality, psychological flexibility, cognitive fusion, and confidence, and that G‐ACT can be performed well by clinical nurses with sufficient training and support.

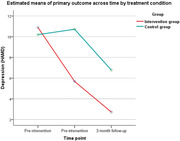

## 1 INTRODUCTION

Expenditure on health care and mental health care is growing and posing serious challenges for governments around the world (Patel et al., 2018). Stroke is not only a leading cause of permanent functional disability, but also often causes severe mental health impairment (Kowalska et al., 2020). Poststroke depression (PSD) is the most frequent and burdensome neuropsychiatric complication after a stroke (Taylor‐Rowan et al., 2019) and has been reported to be associated with greater morbidity, increased mortality risk, and higher health care costs (Guo et al., 2021; Lokk & Delbari, 2010). PSD occurs in 31%–50% of all stroke patients (Ayerbe et al., 2013; Knapp et al., 2020) and also occurs in acute stroke patients (Kowalska et al., 2020). Studies that assessed patients more than once have indicated that a significant proportion of the patients were depressed shortly after the acute event, but 15%–57% had subsequently recovered 1 year later (Ayerbe et al., 2013). A previous study has shown that PSD had a negative impact on the level of disability and survival rates after a stroke among acute stroke patients (Kowalska et al., 2020). But only 5%–11% of those with PSD are recognized early and treated in a timely manner in hospital or other clinical settings (Muller et al., 2021; Swartz et al., 2016). Recent research has reinforced that PSD remains underdiagnosed and undertreated (Bhattacharjee et al., 2018). Thus, it is important and desirable to recognize and treat depression in acute stroke patients appropriately.

Psychological interventions to build mental wellbeing can have a vital role in reducing the pressure and burden of illness (Slade, 2009). van Agteren et al. (2021) review included 419 randomized controlled trials (RCTs) from clinical and nonclinical populations (*n* = 53,288) and found that acceptance and commitment therapy (ACT) was one of the impactful interventions. There is a growing body of RCTs in support of ACT to treat depression across a wide variety of patients, and ACT has been reported to be able to treat depression and improve quality of life (Bai et al., 2020). ACT proposes that depression is an indication that life is unbalanced, and it is a natural aspect of human experience. The core of ACT is behavioral change enabling living according to one's chosen values (A‐Tjak et al., 2018). ACT has many characteristics indicating that it is suitable for stroke patients, such as a focus on an acceptance of unpleasant feelings, because many stroke patients may have disability for many years with accompanying psychological effects (Majumdar & Morris, 2019).

To date, three RCTs have reported the effectiveness of ACT for stroke patients, and both showed benefits. Firstly, Majumdar and Morris (2019) performed an RCT which compared regular treatments for stroke survivors (without the inclusion criterion of depression and the times since the most recent stroke averaged at 13.6 months) with group‐based ACT (G‐ACT) comprising four 2‐h instructional PowerPoint sessions. The results showed a significant reduction in depression and increased self‐assessed health rating and hopefulness for the G‐ACT intervention patients. However, there were no differences identified relating to quality of life, and the sample size was too small to yield conclusive results with only 56 participants and there was not inclusion criterion for depression in this study.

Secondly, Niu et al. (2021) included 104 acute ischemic stroke patients (having the first ever stroke attack, with the onset of less than a week) with those in the intervention group being given G‐ACT treatment (five sessions, 45–55 min/session). The depression scores of those in the intervention group were significantly lower than those of the control group at 1 month (*p* = .018) and 3 months follow‐up (*p* = .001); however, there were no differences in the other two outcomes including degree of neurological deficits and activities of daily living between the two groups. Additionally, the intervention comprised five sessions which has no scientific basis. Again, there was no inclusion criterion for depression in this study.

Thirdly, Wang et al. (2022) included 100 acute cerebral infarction patients (the times since the most recent stroke was not documented) suffering from insomnia, anxiety, and depression, with those in the observation group being given ACT psychological nursing (seven units, the time for each unit was not clear). After 1 month of nursing, the scores of depression were significantly decreased in the observation group (*p* = .001); however, the intervention integrated ACT into nursing measures, and the study aimed to improve insomnia, anxiety, and depression, rather than depression only.

These three RCTs suggest that ACT may have efficacy in reducing depression following stroke. This study aims to test the effectiveness of ACT in a large hospital by implementing G‐ACT for acute stroke patients with depression and to evaluate changes in the quality of life, and sleep quality. To our knowledge, this is the first large trial in an acute stroke sample testing G‐ACT as the treatment of depressive symptoms following stroke.

## 2 METHODS

### 2.1 Design

This was an RCT with a pretest–posttest design and two arms, in which a 4‐week G‐ACT intervention was compared to usual care. The primary and secondary outcomes were collected using interviews before the intervention and immediately after the intervention in hospital, and then again 3 months later (follow‐up assessment) over the telephone, at the outpatient clinic, or during a home visit. The study was approved by the Ethics Committee of PLA Rocket Force Characteristic Medical Center (KY2018005).

### 2.2 Setting

This study was conducted at the PLA Rocket Force Characteristic Medical Center, which is a large hospital with acute stroke in‐patient treatment.

### 2.3 Patients

Acute stroke patients who had been hospitalized at the PLA Rocket Force Characteristic Medical Center were recruited from August 2020 to February 2021. The inclusion criteria were as follows: (1) primary diagnosis of stroke with a confirmation of magnetic resonance imaging or computed tomography, and with the last onset within 2 weeks, (2) aged over 18‐year old, (3) scoring at least 8 on the Hamilton Depression Rating Scale (HAMD or HDRS) scale, (4) being conscious and able to verbally and effectively communicate with the researchers, (5) having primary school education level or above, (6) having signed informed consent, and able to participate in the group intervention on time. Exclusion criteria were as follows: (1) having a history of mental illness (such as bipolar disorder) or dementia, using psychiatric drugs or sedatives within 6 months, or currently using antidepressants, (2) a diagnosis of cognitive impairment as assessed by the Mini–Mental State Examination, (3) receiving a psychological therapy in the previous 3 months, or currently experiencing severe psychotic symptoms, and (4) a history of drug abuse or dependence or any neurological disorder other than a stroke.

We assessed 693 patients for eligibility, of whom 140 were randomized and 70 received treatment within our study. Not meeting inclusion criteria or declining to participate were the main reasons for exclusion from the study.

### 2.4 Randomization

One hundred and forty patients gave written informed consent for this study, based on the Declaration of Helsinki, and were randomly assigned to one of two groups: G‐ACT or control group. Two independent researchers who did not participate in assessing patients took charge of the randomization using the lottery method. Once a number has been selected, that particular number was struck off from the draw box. Sequentially numbered, opaque, sealed, and stapled envelopes were used to store the assignment results. The allocation process was concealed from other researchers who were responsible for assessing participants. The envelopes were opened only after the corresponding patient had completed the baseline assessment.

### 2.5 Intervention

The patients included to the control group received usual care support in hospital, and the patients included to the intervention group received G‐ACT and usual care support. Based on the Hayes and Wilson (1994) and after expert consultation, the G‐ACT treatment in this study comprised seven sessions of 45–60 min and was administered throughout the course of the 4‐week long trial. The ACT sessions were generally held twice a week with each group enrolling 4–8 patients. A patient was excluded from the study if he/she was absent for three sessions, as this was half or less of the full treatment protocol (Johnsen & Friborg, 2015). The ACT in this study was adapted for acute stroke in collaboration with hospitalized patients and caregivers, including reducing the duration of each session, increasing the number of sessions per week, simplifying the language, and the inclusion of stroke‐specific examples. The session‐by‐session outline is set out in Table 1.

**TABLE 1:** Session‐by‐session outline of acceptance and commitment therapy course.

Session	Content
First	Getting to know stroke, from a broader perspective Familiarity and creating therapeutic communication. Encourage patients to share their views about stroke, ask the patients to share where they get their information about stroke, health education, including the risk factors, outcome, and rehabilitation, creating the distinction between actions that are under our control and actions that are controlled by our mind
Second	Embrace everyday life, be content with the present Encourage the patients to express their feelings and thoughts, normalization of negative thoughts, strengthen the positive thoughts, focus on acceptance of unpleasant feelings without trying to avoid or suppress them
Third	Observe yourself and understand yourself By using self‐portrait, labeling, tearing off the label to teach the patients learn to observe and understand themselves, and recognize that “I'm who I am”
Fourth	Be aware of the present moment and embrace it Guide the patients to be aware of the present moment, and encourage them to describe their current feelings and embrace them, and reinforce positive beliefs
Fifth	Identification of individual values Help the patient identify individual values at present and establish a positive attitude to face the current situation
Sixth	Set goals and commit to action Help the patient develop specific goals based on value orientation, and identify targets in line with their current situation and commit to action
Seventh	Learn how to relax, improve sleep quality By using abdominal breathing, relaxing music, soft, and slow language to let the patient relax, help them learn the relaxation technique and can practice it by themselves

The group training sessions were performed and administered by two research nurses. Prior to the study, the 2 nurses completed an online ACT training course including 16 classes in Meilihua eHealth University, and received supervision from 2 clinical psychologists, who had 5 years of experience in ACT therapy and who participated in designing the intervention sessions as primary researchers in the study. Prior to the study, the two nurses delivered three whole G‐ACT intervention sessions with stroke survivors by themselves with supervision by one of the two clinical psychologists, and the results showed good interventional outcome.

### 2.6 Primary outcome

#### 2.6.1 Depression

The HAMD or HDRS (Hamilton, 1986) is the most common, standard, and classic depression rating scale in clinical research and has been used worldwide by researchers and clinicians due to its high reliability and validity in measuring depressive symptoms in stroke patients (Gao et al., 2012; Robinson et al., 1984). The Chinese version (Zheng et al., 1988) has good reliability and validity. We utilized the 24 item HAMD scale to measure the severity of depression symptoms. Each item is scored from 0 to 2 or 0 to 4; if the total score was less than 8, it would not be considered depression; if the total score was greater than 8, mild depression would be considered; if the total score was greater than 20, moderate depression would be considered; and if the total score exceeds 35, it would be considered severe depression.

### 2.7 Secondary outcomes

#### 2.7.1 Health‐related quality of life (HRQoL)

Health‐related quality of life (HRQoL) was assessed using the Short Form Survey Version 2 (SF‐12v2) (Ware et al., 1996), which comprises 12 items being aggregated into 2 health summary scales: physical component summary (PCS) and mental component summary (MCS) ranging from 0 (worst) to 100 (best). The component scores for the SF‐12 are norm‐based, with a mean score of 50 and standard deviation of ±10. A higher score reflects better HRQoL and a lower score, poorer HRQoL. The SF‐12v2 has been found to be a valid and reliable substitute for the SF‐36 (Lam et al., 2013). It takes less than 2 min to complete SF‐12 and has been widely used with stroke patients, and studies have shown that the Chinese version has good reliability and validity (Liao et al., 2016; Yu et al., 2012).

#### 2.7.2 Psychological flexibility

The Acceptance and Action Questionnaire II (AAQ‐II) (Bond et al., 2011) is a seven‐item questionnaire measuring psychological flexibility. The items are scored on a 7‐point Likert scale and the total score ranges from 7 to 49 with a higher score indicating less psychological flexibility. The Chinese version (Cao et al., 2013) has good internal consistency (Cronbach's *α* = .84).

#### 2.7.3 Cognitive fusion

The Cognitive Fusion Questionnaire (CFQ) (Gillanders et al., 2014) is a 9‐item questionnaire measuring cognitive fusion on a 7‐point Likert scale, scores range from 0 to 63 with a higher score indicating that a person is more fused one with one's thoughts. The Chinese version (Zhang et al., 2014) has good internal consistency (Cronbach's *α* = .92).

#### 2.7.4 Sleep quality

The Pittsburgh Sleep Quality Index (PSQI) (Buysse et al., 1989) is a 19‐item questionnaire evaluating subjective sleep quality over the past month. The items are scored on a scale of 0 to 3, with total scores ranging from 0 to 21 and higher scores indicating poorer sleep quality. The Chinese version of PSQI (Lu et al., 2014) has good internal consistency (Cronbach's *α* = .845) and test‐retest reliability (*α* = .994). If the total score was lower than 5, it would be considered very good sleep quality; if the total score was greater than 6, it would be considered good sleep quality; if the total score was greater than 11, it would be considered moderate sleep quality; and if the total score exceeds 16, it would be considered poor sleep quality.

#### 2.7.5 Confidence

The confidence was assessed using the confidence after stroke measure (CaSM) (Horne et al., 2017), which is a 27‐item questionnaire on a 4‐point Likert type scale with total scores ranging from 0 to 81 and higher scores representing higher confidence. The Chinese version (Hu et al., 2018) has good internal consistency (Cronbach's *α* = .974). If the total score was below 36, low confidence was considered; if the total score was lower than 28, very low confidence was considered.

The posttest and follow‐up assessment were administered by a trained research assistant who was unaware of each patient's treatment allocation, and the patients were asked not to reveal to her the types of treatment that they received.

### 2.8 Statistical analyses

SPSS software version 20 was used for data analysis. Pearson *χ* ^2^ and independent samples t tests analyses were used as the statistical methods for comparisons between groups. Repeated measure ANOVA was used for the comparison of the changes of depression before, immediately after and 3 months after the intervention and, considering its significance, the least significant difference post hoc test was used.

## 3 RESULTS

One patient in the control group declined to complete the second assessment before discharge. Of the patients in the intervention group, 9 (12.9%) patients participated in 5 sessions, 21 (30%) patients participated in 6 sessions, and 40 (57.1%) patients participated in all sessions. Finally, 139 patients (91 males, 48 females; age: 61.91 years ±10.363) were included in the final analysis. There were no significant statistical differences in the demographic and clinical characteristics data (gender, age, marital status, educational level, occupation, monthly gross income, residence, medical insurance, stroke hemisphere, prior cerebral infarction, and NIHSS score [*p* > .05, Table 2]) between the two groups, and 138 (99.3%) patients were assessed as having mild depression.

**TABLE 2:** The demographic and clinical characteristics of the subjects (*N* = 139).

Characteristics	Intervention group	Control group	*p*‐Value
	(*N* = 70)	(*N* = 69)	
Male gender	40 (57.1%)	51 (73.9%)	.050
Age	62.21 ± 9.937	61.61 ± 10.843	.732
Marital status			.527
Married	64 (91.4%)	65 (94.2%)	
Others	6 (8.6%)	4 (5.8%)	
Educational level			.971
Primary school education	2 (2.9%)	3 (4.3%)	
High‐school education	45 (64.3%)	44 (63.8%)	
Junior college education	14 (20.0%)	13 (18.8%)	
Bachelor degree or above	9 (12.9%)	9 (13.0%)	
Occupation			.091
Worker	34 (48.6%)	22 (31.9%)	
Cadres	24 (34.3%)	22 (31.9%)	
Soho	3 (4.3%)	6 (8.7%)	
Farmer	5 (7.1%)	14 (20.3%)	
Employee	4 (5.7%)	5 (7.2%)	
Monthly gross income in PRC currency			.594
<1000	2 (2.9%)	5 (7.2%)	
1000–3000	8 (11.4%)	7 (10.1%)	
3000–5000	28 (40.0%)	23 (33.3%)	
≥5000	32 (45.7%)	34 (49.3%)	
Residence			.066
City	63 (90.0%)	54 (78.3%)	
Countryside	7 (10.0%)	15 (21.7%)	
Medical insurance			
With	65 (92.9%)	59 (85.5%)	.183
Without	5 (7.1%)	10 (14.5%)	
Stroke hemisphere			.271
Left	26 (37.1%)	35 (50.7%)	
Right	32 (45.7%)	25 (36.2%)	
Bilateral	12 (17.1%)	9 (13.0%)	
Prior stroke history	39 (55.7%)	32 (46.4%)	.352
NIHSS score	3.97 ± 5.605	3.36 ± 5.099	.504

### 3.1 Primary outcome

There was no significant statistical difference regarding the score of HAMD at pre‐intervention between the two groups (*p* > .05, Table 3). The results demonstrated that the lengthening of time between intervention and follow‐up resulted in a decrease of HAMD scores for both study groups with the ACT group having significantly lower scores compared to the control group at post‐intervention (*p* < .001) and 3‐month follow‐up (*p* < .001, Table 3), suggesting a reduction of depressive symptoms after the intervention. A mixed‐design repeated‐measures ANOVA found a significant time × group interaction for depression, in favor of G‐ACT (Table 4). The partial η^2^ for this effect was .306, which indicates that G‐ACT had an effect on reducing depression, and the effect was still significant over time.

**TABLE 3:** Estimated means of primary outcome across time by treatment condition.

Measure	Time point	Intervention group	Control group	*t*‐Value	*p*‐Value
		(*N* = 70)	(*N* = 69)		
HAMD	T1	10.9 ± 2.644	10.2 ± 2.632	1.668	.122
	T2	5.69 ± 2.130	10.72 ± 3.625	–9.973	<.001
	T3	2.73 ± 2.126	6.78 ± 4.553	–6.710	<.001

*Note*: T1, pre‐intervention; T2, post‐intervention; T3, 3‐month follow‐up.

Abbreviation: HAMD, the Hamilton Depression Rating Scale.

**TABLE 4:** Repeated measurement analysis of variance of depression across time by treatment condition.

			df	Mean square	*F*	Partial η^2^
	Within‐subjects	Time	1.654	1428.323	218.299**	.614
Depression		Time × group	1.654	395.233	60.406***	.306
		Error (time)	226.624	6.543		
	Between‐subjects	Group	1	272.157	46.625***	.254
		Error	137	17.512		

*Note*: Mauchly's test of sphericity (*p* < .001).

***p < .001 indicates acceptance and commitment therapy has effect on reducing depression, and the effect is still significant over time.

### 3.2 Secondary outcomes

There were no significant statistical differences regarding the score of MCS, PSQI, AAQ, CFQ, and CaSM at pre‐intervention between the two groups (*p* > .05, Table 5). There were significant differences across all the scores at post‐intervention and at 3‐month follow‐up between the two groups (*p* < .001), indicating that G‐ACT significantly improved HRQoL‐MCS, sleep quality, psychological flexibility, cognitive fusion, and confidence compared with control group. However, there was no significant difference in the scores of PCS between the two groups at any time point (*p* > .05, Table 5).

**TABLE 5:** Estimated means of secondary outcome across time by treatment condition.

Measure	Time point	Intervention group	Control group	*t*‐Value	*p*‐Value
		(*N* = 70)	(*N* = 69)		
MCS	T1	43.76 ± 10.459	46.07 ± 10.161	–1.323	.188
	T2	52.12 ± 8.236	42.15 ± 8.571	6.945	<.001
	T3	57.16 ± 4.708	47.00 ± 9.864	7.730	<.001
PCS	T1	40.42 ± 10.499	38.49 ± 11.052	1.053	.294
	T2	40.7 ± 10.494	38.54 ± 10.833	1.183	.239
	T3	44.05 ± 11.203	43.99 ± 11.959	0.025	.980
PSQI	T1	8.13 ± 3.375	7.78 ± 3.933	0.557	.579
	T2	5.14 ± 2.510	8.79 ± 3.987	–6.361	<.001
	T3	4.20 ± 2.292	6.62 ± 3.730	–4.558	<.001
AAQ	T1	18.11 ± 8.883	17.29 ± 9.261	0.536	.593
	T2	13.59 ± 5.627	19.10 ± 7.832	–4.740	<.001
	T3	11.43 ± 3.658	17.01 ± 6.045	–6.580	<.001
CFQ	T1	24.57 ± 11.530	22.49 ± 11.077	1.084	.280
	T2	18.81 ± 7.014	25.74 ± 9.528	–4.874	<.001
	T3	16.04 ± 4.947	21.52 ± 7.559	–5.049	<.001
CaSM	T1	46.94 ± 9.638	49.93 ± 9.798	–1.810	.072
	T2	55.20 ± 7.375	46.79 ± 9.950	5.625	<.001
	T3	66.44 ± 7.426	57.12 ± 12.471	5.348	<.001

*Note*: T1, pre‐intervention; T2, post‐intervention; T3, 3‐month follow‐up.

Abbreviations: AAQ, the Acceptance and Action Questionnaire; CaSM, the confidence after stroke measure; CFQ, the Cognitive Fusion Questionnaire; MCS, mental component summary; PCS, physical component summary; PSQI, the Pittsburgh Sleep Quality Index.

## 4 DISCUSSION

This study found that the G‐ACT intervention significantly reduced depression in acute stroke patients when compared with the control group at post‐intervention and this difference persisted at 3 months, which echoes the findings of RCTs using G‐ACT with acute stroke patients (Niu et al., 2021) and stroke survivors (Majumdar & Morris, 2019). However, both studies had small samples and neither included stroke patients with a diagnosis of depression. Although Rauwenhoff et al. (2019) published a protocol of ACT for depressive stroke patients, the results related to that trial have yet to be published. As stroke patients experience both physical and emotional changes in the acute phase, it is very important to provide timely emotional support for them (Niu et al., 2021). Our study testified the efficacy of G‐ACT in reducing the depressive symptoms in patients with acute stroke, which has important implications for researchers and clinical staff.

Most of the included patients were assessed as having mild depression with those having severe psychotic symptoms and who may need individual therapy or more specialized psychiatric treatments excluded from our study. The promising results suggested that G‐ACT was effective in patients with mild depression, which is similar to other reported findings (Bai et al., 2020). Its effectiveness for patients with major depression requires further study as does the suitability of G‐ACT delivered by clinical professionals, including nurses, for patients with major or moderate depression.

This study also found that significant G‐ACT intervention effects were observed for HRQoL‐MCS at post‐intervention which persisted at 3 months in acute stroke patients. However, there was no outstanding difference in the PCS scores between the two groups at any time point. The lack of effect of G‐ACT on PCS in our study was similar to Dewhurst et al. (2015) finding for epilepsy patients but contrary to Lundgren et al.’s (2008) study showing significant improvements in quality of life following a short‐term ACT treatment in epilepsy patients. The process of ACT represents psychological flexibility which has been shown to be important in improving psychological health (Kashdan & Rottenberg, 2010). The lack of effect of G‐ACT on PCS found in our study may due to ACT being primarily focused on emotional, interpersonal, and cognitive difficulties and is, therefore, more effective in improving the mental well‐being aspects of HRQoL rather than physical functioning (Dewhurst et al., 2015). Furthermore, the 3‐month time frame of the follow‐up might have been too short a time gap for such life changes to become observable as a response to the intervention.

This study assessed an ACT intervention change in psychological flexibility, and the findings indicated that G‐ACT could improve psychological flexibility significantly over time, which was similar to a review (Graham et al., 2016) where all six different studies observed significant pre‐to‐post intervention improvements following ACT measures of psychological flexibility with AAQ. The overall purpose of ACT is to increase psychological flexibility, which is the ability to be mindful of experiences in the present moment, in an open and neutral manner, while being with one's values (Levin et al., 2014). This study applied interactive, discursive, and more empirical acceptance‐based strategies that are found within traditional ACT group formats, including encouraging patients to share their views about stroke and discussing it with the other patients or their family, inviting previous patients whom had benefited from ACT and would love to share successful experiences with those new to ACT, and using self‐portrait, labeling, tearing off the labels to teach patients to observe and understand themselves, and recognize that “I'm who I am” which may be a particularly good fit for stroke patient groups because it enables the individual to move forward with life and accept their neurocognitive and physical limitations. The ACT focus on value‐driven strategies may be especially useful in this situation. More studies need to test its efficacy on a wider range of stroke patients.

The group training sessions in the study were delivered by two research nurses suggesting that the G‐ACT intervention can be delivered by non‐psychologists including nurses who provide health care for the longest time to patients. As the psychotherapy is not available for in‐patients in China, the results of our study have important clinical implications because it means nurses or other non‐psychologists can implement ACT for stroke patients to reduce depression or other symptoms. Future multicenter studies need to test this conclusion. The nurses in this study had online and face‐to‐face training from two clinical psychologists and delivered three practice sessions prior to the study, which is similar to Majumdar and Morris (2019) study. Training prior to delivering the intervention is very important to assure the success of the therapy, and it is important to establish a standard protocol for learning the ACT.

### 4.1 Strengths and limitations

To our knowledge, this is the first large‐scale trial which focused on testing the effectiveness of G‐ACT in treating depression for acute stroke patients. The results showed that G‐ACT could reduce depression, and the effect was still significant over time (partial $\eta^{2}=.306$). The G‐ACT in this study was adapted for the acute stroke scenario in collaboration with hospitalized patients and caregivers. The changes included reducing the duration of each session, increasing times per week, simplifying the language, and the inclusion of stroke specific examples. The group training sessions in this study were performed by two research nurses with appropriate training and support suggesting the potential of nurses and other clinical professionals as deliverers of G‐ACT. The study had a high response rate which may reflect the maintenance of good relationships between the researchers and sample.

This study has several limitations. The study was conducted in one center with mainly hospitalized patients with acute stroke whose length of hospitalization was mostly less than 1 month. Although the study increased the occurrences of G‐ACT per week, there were still some patients who were unable to complete all seven sessions at the hospital. Participation in the study was voluntary and excluded patients using psychiatric drugs including antidepressants or sedatives so that the sample may not have been representative of the overall population, and a single site limits the generalizability of the findings. Most of the included patients were assessed as having mild depression, and it might be expected that their mental health would improve along with the recovery of neurological function compared with those with severe or moderate depression (Niu et al., 2021).

## 5 CONCLUSION

This study found that G‐ACT could significantly reduce depressive symptoms in patients with acute stroke, and the interventional efficacy was maintained at 3‐month follow‐up (partial η^2^ for this effect was .306), with similar benefits being observed for HRQoL‐MCS, sleep quality, psychological flexibility, cognitive fusion, and confidence, and that G‐ACT can be performed well by clinical nurses with sufficient training and support. The study findings suggest that G‐ACT is an acceptable and promising intervention following acute stroke. The next logical step for future research in this field would be to test the efficacy of ACT interventions against alternative, suitably “active” treatments, including traditional CBT and pharmacotherapy, and to measure both of the variability of depression and stroke severity in the long term.

## AUTHOR CONTRIBUTIONS

**Yun‐E. Liu**: Supervision; writing—original draft. **Jin Lv**: Conceptualization. **Fangzhen Sun**: Data curation; investigation. **Jingjing Liang**: Investigation. **Ying‐Ying Zhang**: Formal analysis. **Jie Chen**: Funding acquisition; project administration. **Weijian Jiang**: Methodology; project administration.

## CONFLICT OF INTEREST STATEMENT

The authors declare that they have no conflicts of interest.

## FUNDING INFORMATION

Beijing Municipal Science & Technology Commission (Grant Number: Z181100001718041)

## PATIENT CONSENT STATEMENT

All included patients gave written informed consent for this study, based on the Declaration of Helsinki.

## CLINICAL TRIAL REGISTRATION

https://register.clinicaltrials.gov. Unique identifier: NCT05727358.

### PEER REVIEW

The peer review history for this article is available at https://publons.com/publon/10.1002/brb3.3260.

## Data Availability

The datasets used and/or analyzed during the current study are available from the first author on reasonable request. The funding agency demands that the data cannot be shared publicly until 3 years after it has been collected.
